# The role of prosocial tendencies in the relationships between gratitude, perceived social support, and psychological well-being among Chinese university students: a structural equation modeling approach

**DOI:** 10.3389/fpsyg.2025.1510543

**Published:** 2025-01-29

**Authors:** Xu Man, Zhang Jing

**Affiliations:** ^1^School of General Courses, Jiangsu Medical College, Jiangsu, Yancheng, China; ^2^Baoding Vocational and Technical College, Baoding, China

**Keywords:** gratitude, perceived social support, prosocial tendencies, psychological well-being, structural equation modeling, university students, mediation, gender invariance

## Abstract

**Introduction:**

This study explores the relationships among gratitude, perceived social support, prosocial tendencies, and psychological well-being (PWB) in Chinese university students. Despite growing interest in these constructs, the mechanisms through which gratitude and social support influence PWB remain underexplored, particularly in collectivist cultural contexts where social harmony and interdependence are prioritized.

**Methods:**

Data were collected from 703 Chinese university students using validated instruments measuring gratitude, perceived social support, prosocial tendencies, and psychological well-being. Structural equation modeling (SEM) was employed to test the direct and indirect effects of gratitude and social support on PWB, with prosocial tendencies modeled as a mediating higher-order factor. Multi-group SEM analysis was conducted to examine gender invariance.

**Results:**

Gratitude and perceived social support significantly predicted PWB, both directly and indirectly through prosocial tendencies. Gratitude had a strong positive effect on prosocial tendencies (*β* = 0.412, *p* < 0.001), which, in turn, significantly contributed to PWB (*β* = 0.465, *p* < 0.001). Similarly, perceived social support positively influenced prosocial tendencies (*β* = 0.375, *p* < 0.001) and PWB (*β* = 0.253, *p* < 0.05). Mediation analysis confirmed that prosocial tendencies partially mediated these relationships. Multi-group SEM analysis revealed structural invariance across gender.

**Discussion:**

The findings underscore the cultural relevance of gratitude and social support within Chinese society, emphasizing the role of collectivist values in fostering PWB. Practical implications include implementing gratitude-focused interventions and enhancing peer support systems within university mental health programs to promote resilience and well-being among students.

## 1 Introduction

Psychological well-being (PWB) is increasingly recognized as a critical determinant of mental health in university settings, where students face significant academic and social pressures (Harding et al., 2019; He et al., 2018). Defined by eudaimonic principles, PWB encompasses dimensions such as personal growth, positive relationships, and life purpose, which are linked to cognitive functioning, resilience, and academic motivation (Ryff and Keyes, 1995; Ryff, 2013). While mental health challenges like stress and anxiety are well-documented among university students, there is a growing need to identify comprehensive strategies that enhance PWB and mitigate these risks (Morales-Rodríguez et al., 2020).

Research highlights the importance of factors like gratitude, perceived social support, and prosocial tendencies in promoting well-being, but their combined effects remain underexplored (Emmons and McCullough, 2003; Zimet et al., 1988). Gratitude is widely associated with improved emotional regulation and social relationships, while perceived social support is a known buffer against stress and a predictor of resilience (Cohen and Wills, 1985; Froh et al., 2009). Prosocial tendencies, encompassing altruistic and cooperative behaviors, have been linked to a greater sense of purpose and stronger social bonds, particularly in challenging academic environments (Huppert, 2009; Feng and Zhang, 2021). However, limited research has examined how these factors interact to influence PWB within a unified framework, leaving gaps in our understanding of their integrated effects.

This study investigates the mediating role of prosocial tendencies in the relationships between gratitude, perceived social support, and PWB among university students. Unlike prior research that often examines these variables independently, this study adopts a holistic approach to explore their interplay, providing a more comprehensive understanding of the mechanisms underlying well-being in academic contexts. The novel focus on prosocial tendencies as a mediator highlights their role as pathways through which gratitude and social support enhance psychological outcomes (Martela and Ryan, 2016; Wan et al., 2023). Targeting university students—a group uniquely vulnerable to academic stress—this study offers valuable insights into promoting PWB in educational settings. Challenges such as academic overload, financial pressures, and significant life transitions heighten students’ susceptibility to stress-related psychological issues (He et al., 2018; Hernández-Torrano et al., 2020; Sun et al., 2020), emphasizing the need for targeted interventions to support their well-being.

## 2 Literature review and theoretical perspectives

### 2.1 Psychological well-being

Psychological well-being (PWB) is a multidimensional concept encompassing personal growth, autonomy, and positive relationships (Ryff and Keyes, 1995; Schmitt et al., 2014). It emphasizes realizing one’s potential and leading a meaningful life (Houben et al., 2015; Huppert, 2009; Ryff, 2013), contrasting with hedonic well-being, which focuses on happiness and pleasure. In the context of education, PWB influences not only emotional well-being but also cognitive functioning, motivation, and academic performance (Huppert, 2009; Ryff, 2013). University students, who face significant academic demands and life transitions, are particularly vulnerable to fluctuations in PWB (Jackman and Sisson, 2022).

Because of its connection to academic outcomes and resilience, PWB is crucial for university students (Mustafa et al., 2020). Higher PWB not only leads to better academic motivation, emotional regulation, and adaptive coping strategies (Greenier et al., 2021; Li and Hasson, 2020; Tan et al., 2021), but it also strengthens social connections, enabling students to form and maintain meaningful relationships (Tan et al., 2021). Moreover, the COVID-19 pandemic further highlighted PWB’s importance, as students with higher PWB exhibited greater academic persistence and emotional stability during this time of uncertainty (Sun et al., 2020; Morales-Rodríguez et al., 2020).

Several factors predict PWB in university students, including resilience, perceived social support, emotional intelligence, and self-efficacy. Resilience, or the ability to recover from adversity, is critical in helping students manage the emotional and academic stress they encounter (Johnson et al., 2019; Tan et al., 2021). Furthermore, social support from peers, family, and educators enhances emotional well-being by providing a buffer against depressive symptoms and fostering a sense of belonging (Costa et al., 2013; Rueger et al., 2016). Emotional intelligence, which involves the ability to effectively manage emotions, also contributes to PWB by strengthening interpersonal relationships and supporting emotional regulation (Shengyao et al., 2024).

Self-efficacy, or the belief in one’s ability to succeed, is another key predictor of PWB. Students with high self-efficacy tend to be more persistent in the face of academic challenges and exhibit better psychological adjustment to stress (Costa et al., 2013). This relationship was particularly evident during the COVID-19 pandemic, as students with greater self-efficacy, resilience, and social support were better able to maintain their PWB despite the disruptions to their academic and social lives (Marler et al., 2024). Consequently, educational environments that actively foster self-efficacy play a crucial role in supporting students’ well-being and academic success (Tang and Zhu, 2024).

In addition to these individual factors, environmental elements, such as academic stress, financial pressures, and the overall campus environment, can significantly affect students’ PWB (Mazzucchelli and Purcell, 2015). High levels of academic stress, particularly when combined with insufficient institutional support, have been linked to lower well-being and increased psychological distress (Morales-Rodríguez et al., 2020). Conversely, universities that provide accessible mental health resources and create inclusive, supportive environments can enhance students’ well-being, leading to improved academic performance and emotional stability (Hernández-Torrano et al., 2020; Huppert, 2009). These findings highlight the important role that institutions play in shaping students’ well-being by addressing both their academic and emotional needs.

In conclusion, PWB is a multifaceted construct crucial to the academic and personal success of university students, affecting not only academic outcomes but also broader aspects of their social and emotional functioning. By focusing on key predictors such as resilience, social support, emotional intelligence, and self-efficacy, educational institutions can develop strategies to enhance students’ well-being and help them navigate the complexities of higher education. Ultimately, the role of institutions in creating supportive environments that address both academic and emotional needs is essential for promoting long-term PWB and academic achievement.

### 2.2 Gratitude and its role in enhancing well-being

Gratitude, a core concept in positive psychology, refers to the recognition and appreciation of the positive aspects of life and the contributions of others (Emmons and McCullough, 2003; Gulliford and Morgan, 2017). It can be experienced both as a dispositional trait and a momentary emotional state, each of which enhances well-being by fostering positive affect and strengthening social connections (Emmons and Shelton, 2001). According to Fredrickson’s (2004) broaden-and-build theory, gratitude expands individuals’ cognitive and behavioral repertoires, building lasting personal resources. This approach aligns with the eudaimonic view of well-being, which emphasizes personal growth and meaningful social engagement over short-term pleasure (Wood et al., 2009). Gratitude not only predicts life satisfaction but also enhances psychological well-being (PWB) beyond the influence of other personality traits like the Big Five (Watkins, 2013; Wood et al., 2009).

Gratitude is increasingly recognized as a critical contributor to life satisfaction, happiness, and PWB (Datu, 2014). Ryff and Keyes (1995) conceptualization of PWB includes dimensions such as autonomy, personal growth, and positive relationships, all of which are positively influenced by gratitude. By promoting positive affect, gratitude supports a more optimistic and growth-oriented perspective, which reinforces these dimensions of PWB. Studies consistently show that gratitude is linked to greater resilience, improved emotional regulation, and better coping with stress (Demichelis et al., 2024; Portocarrero et al., 2020). In educational settings, gratitude plays a particularly important role in buffering against stress, thereby enhancing PWB among students (Deichert et al., 2021). Bono et al. (2022) highlight that students who cultivate gratitude experience greater academic success, stronger social relationships, and better emotional health.

The importance of gratitude in well-being became even more evident during crises like the COVID-19 pandemic. Research revealed that students who practiced gratitude were better able to handle the stress of isolation, academic disruptions, and uncertainty (Tolcher et al., 2024). Meta-analyses confirm that dispositional gratitude correlates with higher psychological well-being, greater life satisfaction, and reduced depressive symptoms across diverse populations (Portocarrero et al., 2020). Mason’s (2019) longitudinal study of South African students showed that gratitude acted as a buffer against anxiety and depression, further illustrating its universal benefits.

Gratitude interventions, such as journaling and reflective practices, have shown significant improvements in mental health. Tolcher et al. (2024) found that these interventions led to sustained increases in well-being and reductions in stress by shifting attention from negative to positive experiences, fostering psychological resilience (Dickens, 2017; Wood et al., 2009). Similarly, Zhang et al. (2023) found that gratitude practices increased well-being and reduced emotional exhaustion in Chinese young adults. Several mechanisms explain this link. Gratitude encourages positive cognitive reappraisal, reinterpreting negative events by focusing on benefits or lessons learned, enhancing emotional regulation and stress management (Fredrickson, 2004; Deichert et al., 2021). It also fosters prosocial behavior by increasing empathy and a sense of obligation towards others, strengthening social bonds (Emmons and McCullough, 2003; Kardas et al., 2019). This creates a reinforcing cycle of well-being (Fredrickson, 2004). Gratitude promotes emotional resilience by encouraging a focus on positive aspects, even in difficult situations (Arnout and Almoied, 2021). Huang et al. (2020) found that gratitude moderated the relationship between academic stress and psychological health in students. Finally, gratitude fosters an internal locus of control, enhancing self-efficacy and supporting goal achievement (Portocarrero et al., 2020).

Taken together, gratitude is a powerful resource for enhancing psychological well-being. Its positive effects are mediated through cognitive reappraisal, prosocial behavior, emotional resilience, and an internal locus of control. These findings highlight the importance of incorporating gratitude into mental health interventions and educational programs to improve student well-being across different contexts and populations.

### 2.3 The importance of perceived social support in PWB

Perceived social support—an individual’s belief in the availability of help from their social network (Procidano and Smith, 1997; Zimet et al., 1988)—differs from objective support, which focuses on actual help received. Perceived support often has a stronger impact on psychological outcomes (Zimet et al., 1988). The Multidimensional Scale of Perceived Social Support (MSPSS) (Zimet et al., 1988) categorizes support into three sources: family, friends, and significant others, each offering distinct types of assistance. The belief in their availability shapes an individual’s well-being (Tonsing et al., 2012).

Perceived social support’s benefits are well-established. Research shows that perceiving supportive relationships significantly improves psychological outcomes, often exceeding the effects of actual support (Feeney and Collins, 2015). Individuals with high levels of perceived support report greater life satisfaction, more positive affect, and lower levels of depression and anxiety (Malkoç and Yalçın, 2015). This is particularly relevant for students facing academic stress. Cohen and Wills’ (1985) buffering hypothesis suggests that social support mitigates stress’s negative effects by providing emotional and practical resources.

In educational settings, perceived social support plays a critical role in enhancing emotional regulation and psychological resilience (Hou et al., 2024). Students who feel supported by their social networks are more likely to experience personal growth, autonomy, and environmental mastery, all of which contribute to well-being (Derakhshan et al., 2024; Malkoç and Yalçın, 2015). Furthermore, social support fosters the development of effective coping strategies, allowing students to better navigate academic and interpersonal challenges (Wang et al., 2023). This is particularly significant for university students transitioning to new academic and social environments, where feelings of isolation can negatively affect their well-being (Tomás et al., 2020).

Empirical studies confirm the link between perceived social support and psychological resilience. Malkoç and Yalçın (2015) found that higher perceived support predicted greater resilience and improved well-being in students. Emadpoor et al. (2016) showed that academic motivation mediated the relationship between social support and well-being, suggesting support networks enhance intrinsic motivation. This is particularly critical for international students facing cultural adjustment challenges. Brunsting et al. (2021) found that higher perceived support in international students led to greater emotional well-being and reduced homesickness.

Several theoretical models explain how social support influences well-being. The stress-buffering model proposes that social support mitigates stress by providing resources (Cohen and Wills, 1985). This is relevant for students facing academic pressures, as support helps them manage stress (Lai and Ma, 2016). The main effects model suggests a direct influence of social support on well-being, independent of stress (Lee and Goldstein, 2016). This model emphasizes the intrinsic value of social relationships for fostering belonging and self-worth (Li and Jiang, 2018). In university students, support from peers, family, and faculty enhances competence and social integration (Rehman et al., 2020). Bandura’s (1986) social cognitive theory posits that perceived support enhances self-efficacy, promoting emotional resilience and well-being (Guzman Villegas-Frei et al., 2024; Yildirim et al., 2023).

In summary, perceived social support is a vital determinant of psychological well-being, particularly in student populations. It acts as a protective factor by buffering against stress, enhancing emotional stability, and fostering a sense of belonging. Theoretical frameworks such as the stress-buffering model, the main effects model, and social cognitive theory provide valuable insights into the mechanisms through which social support positively impacts PWB. Understanding these pathways highlights the importance of fostering supportive social environments in educational settings to promote well-being and academic success.

### 2.4 Prosocial behavior and its psychological benefits

Prosocial tendencies encompass behaviors like helping, sharing, and cooperating, driven by intrinsic motivation, empathy, and social norms (Carlo and Randall, 2002; Guan et al., 2019). These actions, initially conceptualized as selfless and other-oriented (Eisenberg, 1989), include various forms serving distinct social and psychological functions (Carlo and Randall, 2002; Eberly-Lewis and Coetzee, 2015). The Prosocial Tendencies Measure (PTM) captures this complexity by assessing a range of prosocial behaviors, from public altruism to private, self-sacrificial acts (Carlo and Randall, 2002). A strong link exists between prosocial behavior and psychological well-being. Engaging in these behaviors generates positive emotions, strengthens social bonds, and enhances a sense of purpose and self-worth (Helliwell et al., 2018; Martela and Ryan, 2016), creating a positive feedback loop. Fredrickson’s broaden-and-build theory (2004) explains how positive emotions from prosocial acts expand individuals’ thought-action repertoires, leading to the development of personal and social resources that contribute to higher life satisfaction and reduced stress (Feng and Zhang, 2021).

Empathy is central to driving prosocial behavior by enabling individuals to emotionally connect with others’ needs (Decety et al., 2016). Individuals with higher levels of empathy are more likely to help others, which strengthens their social bonds and emotional well-being (Telle and Pfister, 2016). Additionally, emotional regulation enhances prosocial behavior by allowing individuals to manage their emotions effectively, helping them respond constructively to others’ needs (Bailey et al., 2020; Sharma and Tomer, 2018). Positive emotions, such as gratitude and compassion, further encourage prosocial behavior by fostering a sense of interconnectedness and moral responsibility, benefiting both the helper and the recipient (Aknin et al., 2018).

Social support also plays a key role in fostering prosocial behavior. Individuals who perceive strong social support are more likely to engage in helping behaviors, as supportive relationships provide a sense of security, belonging, and reciprocity (Fu et al., 2022; Rosli and Perveen, 2021). This reciprocal relationship strengthens social networks, enhancing emotional well-being for both the giver and the receiver (Haller et al., 2022). In both educational and organizational settings, fostering positive social environments is essential, as strong interpersonal relationships promote collective well-being and encourage prosocial tendencies (Collie, 2022; Martela and Ryan, 2016).

The development of prosocial tendencies is significantly influenced by environmental factors, particularly during childhood and adolescence (Knafo and Plomin, 2006). Supportive and emotionally nurturing environments in schools and families encourage prosocial behavior in young people (Su et al., 2021). When students’ needs for relatedness are met, particularly in educational settings, their motivation to engage in prosocial acts increases, promoting psychological well-being (Collie, 2022; Liu et al., 2021). These findings highlight the importance of social–emotional learning initiatives that cultivate empathy, emotional intelligence, and social responsibility, fostering personal growth and encouraging positive contributions to others (Martela and Ryan, 2016).

Overall, prosocial tendencies are closely linked to psychological well-being. Empathy, emotional regulation, and social support are key drivers of prosocial behavior, enhancing individuals’ sense of purpose, belonging, and emotional resilience. Given the reciprocal relationship between prosocial behavior and well-being, promoting these behaviors not only benefits those on the receiving end but also enhances the mental health of those who engage in them. Fostering prosocial behavior is therefore essential for improving individual well-being and strengthening social cohesion.

### 2.5 The study rationale

This study draws on key theoretical frameworks to explain the relationships between gratitude, social support, prosocial behavior, and psychological well-being (PWB). Fredrickson’s broaden-and-build theory (2004) posits that positive emotions, such as gratitude, expand cognitive and behavioral capacities, fostering prosocial behaviors that enhance social bonds and improve PWB (Fredrickson, 2004; Huppert, 2009; Wood et al., 2009). Gratitude not only boosts emotional states but also promotes personal growth and social capital through altruism and cooperation (Emmons and McCullough, 2003). Similarly, Bandura’s social cognitive theory (1986) highlights the role of self-efficacy in encouraging individuals with strong social support to engage in prosocial behaviors, enhancing competence and belonging (Rehman et al., 2020; Schwarzer and Knoll, 2007).

The stress-buffering model (Cohen and Wills, 1985) emphasizes that social support reduces stress, promoting resilience and mental health (Wang et al., 2018). Together, these frameworks suggest that gratitude and social support improve PWB by encouraging prosocial actions, which build resilience, strengthen social connections, and foster purpose (Helliwell et al., 2018; Martela and Ryan, 2016).

Empirical evidence supports these links. Gratitude predicts PWB beyond personality traits and strengthens social bonds, enhancing perceived social support and facilitating prosocial behavior (Portocarrero et al., 2020; Feng and Zhang, 2021; Deichert et al., 2021). Social support, both perceived and actual, plays a vital role in reducing stress, particularly for students managing academic pressures (Brunsting et al., 2021; Costa et al., 2013). Students with greater social support report improved emotional well-being, reduced homesickness, and better psychological health (Li et al., 2023). Prosocial behaviors, such as helping others, foster relatedness, competence, and resilience, directly contributing to PWB (Huppert, 2009; Wan et al., 2023). Gratitude and social support promote prosociality by fostering empathy and reciprocity, which strengthen social ties and well-being (Emmons and McCullough, 2003; Su et al., 2021). Prosocial actions also help individuals build psychological resources like emotional regulation, buffering against stress and enhancing mental health (Haller et al., 2022; Zhang et al., 2023). Research demonstrates that prosocial behavior mediates the relationship between social support and mental health, as well as between gratitude and PWB (Li et al., 2023). This highlights the importance of prosociality in translating gratitude and social support into psychological benefits (Deichert et al., 2021; Zhang et al., 2023).

To address these gaps, this study seeks to answer the following research question: *How do gratitude and perceived social support, both directly and indirectly via prosocial tendencies, influence psychological well-being among Chinese university students?*

The primary objective of this study is to examine the mediating role of prosocial behavior in the relationships between gratitude, perceived social support, and psychological well-being. Specifically, the research aims to identify the mechanisms through which gratitude and social support contribute to psychological health and to test whether these mechanisms are consistent across gender groups.

Based on these theoretical frameworks and empirical evidence, the following hypotheses are proposed:

*Hypothesis 1*: Gratitude will be positively associated with psychological well-being.

*Hypothesis 2*: Perceived social support will be positively associated with psychological well-being.

*Hypothesis 3*: Prosocial behavior will mediate the relationship between gratitude and psychological well-being, such that individuals with higher levels of gratitude will engage in more prosocial behaviors, which in turn will enhance their psychological well-being.

*Hypothesis 4*: Prosocial behavior will mediate the relationship between perceived social support and psychological well-being, such that individuals who perceive higher levels of social support will engage in more prosocial behaviors, which will lead to greater psychological well-being.

## 3 Research design and methodology

### 3.1 Sample characteristics

A total of 703 university students from various academic disciplines in China participated in this study. University students represent a particularly relevant population for this study for several reasons. First, they are at a critical developmental stage marked by significant transitions and challenges that can impact their mental health and well-being. Second, university students are often exposed to high levels of academic stress and social pressures, making them particularly vulnerable to psychological distress (Li and Hasson, 2020). Third, this population is increasingly reliant on social connections and support networks to navigate these challenges, making it crucial to understand the factors that contribute to their psychological well-being in a university context. Furthermore, examining these relationships within a Chinese context provides valuable insights into how gratitude, social support, and prosocial tendencies operate within a collectivist cultural framework.

The participants were recruited over a three-month period, from March to May 2023, using a combination of online and in-person strategies to ensure a diverse and representative sample. The participants ranged in age from 18 to 25 years (*M* = 21.3, SD = 1.7), with 56% identifying as female (*n* = 394) and 44% as male (*n* = 309). The majority of participants were undergraduate students (83%), while the remaining 17% were enrolled in graduate programs.

The sample included participants from various socioeconomic backgrounds, ranging from low-income families to those with higher household incomes. Based on self-reported parental education levels, approximately 35% of participants came from families where neither parent held a college degree, while 42% reported at least one parent with higher education, and 23% indicated both parents had advanced degrees. This distribution highlights the socioeconomic diversity within the sample. Geographically, students were drawn from 15 provinces across China, with a majority (68%) coming from urban areas and 32% from rural regions. This geographic spread ensures representation from diverse cultural and environmental contexts, capturing differences in regional access to educational resources and social support networks. Additionally, participants represented a wide array of academic disciplines, including humanities (26%), sciences (22%), engineering (18%), business (16%), and medical fields (18%). This variety provides a comprehensive view of the student population, avoiding overrepresentation of any single academic focus.

The participants were required to be full-time university students in China. Exclusion criteria included part-time student status or a history of psychiatric disorders that could affect responses to psychological well-being measures. The final sample represented a diverse range of socioeconomic and academic backgrounds, providing a broad cross-section of the Chinese student population. Ethical approval was obtained from the Department of Basic Education at Jiangsu Medical College. Participants were informed about the study’s purpose, assured of voluntary participation, and provided with informed consent forms online before beginning the survey. Anonymity was guaranteed, and participants could withdraw at any time without consequence.

To enhance generalizability, students were recruited from multiple academic disciplines, including humanities, sciences, engineering, and medical fields. Participation was voluntary, with no monetary incentives offered. Contact information for psychological support services was provided to address any emotional discomfort arising from the survey.

## 4 Instruments

### 4.1 Gratitude

The disposition to experience and express gratitude was measured using the Gratitude Questionnaire-6 (GQ-6), developed by McCullough et al. (2002). This six-item scale assesses individual differences in gratitude proneness. Participants responded to items like “I have a lot to be thankful for in my life” on a 7-point Likert scale (1 = strongly disagree to 7 = strongly agree). A higher average score indicates a greater tendency towards gratitude. The GQ-6 was translated into Chinese following a standardized translation and back-translation protocol. Bilingual experts reviewed the translated version to ensure semantic and conceptual equivalence with the original English version.

In the present study, the GQ-6 exhibited satisfactory internal consistency, with a Cronbach’s alpha of 0.83 for the Chinese sample. To further examine the reliability of the GQ-6, we calculated McDonald’s omega (*ω*), which was found to be 0.84. Confirmatory factor analysis (CFA) for the GQ-6 demonstrated good construct validity (χ^2^/df = 2.45, CFI = 0.95, TLI = 0.93, RMSEA = 0.06 [0.04, 0.08], SRMR = 0.04), indicating that the items loaded well onto a single factor of gratitude. Convergent validity was assessed by examining the average variance extracted (AVE), which was 0.60, suggesting that the items explain a substantial proportion of the variance in the gratitude construct. Discriminant validity was assessed using the Fornell-Larcker criterion, which compares the AVE for each construct to the squared correlations between constructs. The results indicated that the AVE for gratitude was greater than its squared correlations with other constructs in the model (see Table 1), supporting discriminant validity.

**Table 1:** Discriminant validity of constructs.

Construct	AVE	Gratitude	Perceived social support	Prosocial tendencies	Psychological well-being
Gratitude	0.60	1.00	0.21	0.14	0.23
Perceived social support	0.63	0.21	1.00	0.30	0.18
Prosocial tendencies	0.65	0.14	0.30	1.00	0.17
Psychological well-being	0.64	0.23	0.18	0.17	1.00

Average Variance Extracted (AVE) for each construct (shown on the diagonal) is greater than the squared correlations with all other constructs (shown off-diagonal).

### 4.2 Perceived social support

The perception of social support received from various sources was assessed using the Multidimensional Scale of Perceived Social Support (MSPSS), created by Zimet et al. (1988). This 12-item instrument evaluates perceived support from family, friends, and significant others. Participants rated statements like “I get the emotional help and support I need from my family” on a 6-point Likert scale (1 = strongly disagree to 6 = strongly agree). A higher average score signifies stronger perceived social support. The MSPSS was translated into Chinese using a standardized translation and back-translation procedure, followed by a review by bilingual experts to ensure the semantic and conceptual equivalence of the translated items.

To assess the internal consistency of the MSPSS, we calculated Cronbach’s alpha, which was found to be 0.87, indicating excellent reliability. Additionally, McDonald’s omega was calculated for each subscale of the MSPSS, with values ranging from 0.81 to 0.86, further supporting the internal consistency of the measure. CFA results for the MSPSS indicated a good fit (χ^2^/df = 3.10, CFI = 0.93, TLI = 0.91, RMSEA = 0.07 [0.05, 0.09], SRMR = 0.05), suggesting that the three-factor structure (family, friends, significant others) is a good representation of the data. Convergent validity was supported with an AVE of 0.63, indicating that the items explain a substantial amount of variance in the perceived social support construct. Discriminant validity checks showed that the AVE for perceived social support exceeded the squared correlations with other constructs in the model (see Table 1).

### 4.3 Prosocial tendencies

The inclination to engage in helpful behaviors towards others was measured with the Prosocial Tendencies Measure (PTM), developed by Carlo and Randall (2002). This 26-item scale comprises six subscales: public, anonymous, altruistic, compliant, emotional, and urgent, each capturing a different facet of prosocial behavior. Respondents indicated their agreement with items such as “I feel that helping others is a way to gain people’s approval” on a 5-point Likert scale (1 = strongly disagree to 5 = strongly agree). The PTM was adapted into Chinese using a standardized translation and back-translation protocol. A committee of bilingual experts in psychology reviewed the translated version to ensure that the meaning and nuances of each item were accurately captured in Chinese.

The PTM exhibited good internal consistency in the current study, with a Cronbach’s alpha of 0.81. Internal consistency for each subscale was assessed, with Cronbach’s alpha values ranging from 0.72 to 0.84, indicating acceptable to good reliability for all dimensions. Specifically, the alpha values were as follows: altruistic (0.84), compliant (0.77), emotional (0.79), public (0.72), anonymous (0.80), and urgent (0.81). McDonald’s omega (*ω*) was also calculated for each subscale, with values all exceeding 0.75, providing further evidence of good reliability. Confirmatory factor analysis for the PTM subscales showed acceptable construct validity (χ^2^/df = 2.80, CFI = 0.91, TLI = 0.89, RMSEA = 0.07 [0.06, 0.09], SRMR = 0.05). All factor loadings were statistically significant (*p* < 0.001), and the model fit indices support the six-factor structure of the PTM. In our study, prosocial tendencies were modeled as a higher-order latent construct to capture the shared variance among these dimensions while maintaining parsimony and interpretability in the structural equation model. This higher-order factor showed excellent internal consistency, with ω = 0.88. Convergent validity was indicated by AVEs above 0.50 for all subscales, suggesting that the items within each subscale converge to measure a specific dimension of prosocial tendencies. Discriminant validity was confirmed using the Fornell-Larcker approach, which showed that the AVE for each subscale was greater than the squared correlations with other subscales (see Table 1).

### 4.4 Psychological well-being

The overall state of psychological health was evaluated using the 18-item version of the Ryff Psychological Well-being Scale (Ryff and Keyes, 1995). This shortened version of the scale encompasses six dimensions of well-being: autonomy, environmental mastery, personal growth, positive relations with others, purpose in life, and self-acceptance. Participants rated statements like “I feel I am in charge of the situation in which I live” on a 7-point Likert scale (1 = strongly disagree to 7 = strongly agree). A higher mean score across the six dimensions reflects greater psychological well-being. The Ryff scale was translated into Chinese using a rigorous translation and back-translation process, with independent translations by two bilingual individuals. Any discrepancies were resolved through discussion and consensus, and the final translated version was reviewed by a panel of experts to ensure accuracy and cultural appropriateness.

The scale showed high internal consistency in this study, with a Cronbach’s alpha of 0.88. Furthermore, McDonald’s omega for the overall scale was 0.89, and each dimension showed ω values above 0.80, indicating excellent internal consistency. CFA for the Ryff Psychological Well-being Scale confirmed construct validity (χ^2^/df = 3.00, CFI = 0.92, TLI = 0.90, RMSEA = 0.07 [0.06, 0.09], SRMR = 0.05). All factor loadings were statistically significant (p < 0.001), and the model fit indices support the six-factor structure of the Ryff scale. Construct validity was further supported by AVE = 0.64, suggesting that the items explain a substantial amount of variance in the psychological well-being construct. Discriminant validity testing indicated that each dimension captured a distinct facet of well-being, as the AVE for each dimension was greater than the squared correlations with other dimensions (see Table 1).

### 4.5 Procedure

To accommodate participant preferences and enhance engagement, data collection was conducted from March to May 2023 using both online and in-person methods. Recruitment was facilitated through a variety of channels, including university-wide emails, digital flyers, social media announcements, and outreach to academic departments. Participants had the option to complete the survey online via a secure Qualtrics link or in person at designated university locations. In-person surveys were conducted in computer labs under the supervision of research assistants, and both methods used identical survey content and presentation to ensure consistency.

Before starting the survey, participants received an overview of the study’s objectives and procedures. Informed consent was obtained electronically for online participants and via signed forms for in-person participants. To ensure voluntary participation, confidentiality was emphasized, with no identifying information collected, and participants were informed that they could withdraw at any time without penalty. No compensation was offered.

To minimize order effects, survey items were randomized. Standardized questionnaires were used to measure gratitude, perceived social support, prosocial tendencies, and psychological well-being. The survey took approximately 15–20 min to complete, with an option to save progress and return later. Participant well-being was prioritized throughout the data collection process. Upon completing the survey, participants were debriefed and given detailed information about the study, including contact details for the research team. Furthermore, participants who experienced any discomfort were informed of available university counseling services and external mental health resources, and research assistants were trained to address immediate concerns during in-person sessions.

### 4.6 Research team composition and collaborative process

This study was carried out by two principal investigators, one specializing in educational psychology and the other focusing on quantitative data analysis. They collaborated closely in designing the research framework, developing the survey instruments, and interpreting the results. A team of four trained research assistants supported data collection and participant recruitment under the investigators’ supervision. The principal investigators held weekly meetings to discuss recruitment progress, address logistical challenges, and review preliminary findings. This regular communication ensured that all team members contributed to refining the research design and maintaining data quality. After data collection, both investigators jointly analyzed the results and worked together on writing and revising the manuscript, integrating feedback from all team members to ensure accuracy and coherence.

### 4.7 Data analysis

Data analysis was conducted using structural equation modeling (SEM) with AMOS 26.0, complemented by assumption testing and data screening in SPSS 26.0. First, to ensure the quality of the data, we conducted preliminary analyses. This included assessing normality through skewness and kurtosis values, multicollinearity via Variance Inflation Factor (VIF) values (Hair et al., 2010), and homoscedasticity by inspecting residual scatter plots. Outliers were identified using univariate and multivariate methods and were excluded to ensure the robustness of the findings.

Next, we performed a confirmatory factor analysis (CFA) to assess the construct validity of our measurement model. This involved examining the fit indices including Comparative Fit Index (CFI), Tucker-Lewis Index (TLI), Root Mean Square Error of Approximation (RMSEA), and Standardized Root Mean Square Residual (SRMR), following Hu and Bentler (1999) criteria. Good model fit was defined by CFI and TLI values above 0.90, RMSEA below 0.08, and SRMR below 0.08 (Kline, 2016).

Following the CFA, we evaluated the reliability and convergent validity of the measures. We assessed reliability using both Cronbach’s alpha and McDonald’s omega, with values above 0.70 for Cronbach’s alpha and 0.80 for McDonald’s omega considered to indicate good internal consistency. Convergent validity was evaluated by examining the factor loadings of the items on their corresponding latent variables, with high loadings indicating that the items are measuring the same construct. We also calculated the average variance extracted (AVE) for each construct, with AVE values above 0.50 generally considered to indicate good convergent validity (Fornell and Larcker, 1981).

Subsequently, we assessed discriminant validity using the Fornell-Larcker criterion, which compares the AVE for each construct to the squared correlations between constructs. Discriminant validity is supported when the AVE for each construct is greater than the squared correlations with other constructs (Henseler et al., 2015). The heterotrait-monotrait (HTMT) ratio of correlations was also examined as a further test of discriminant validity.

Finally, we conducted structural equation modeling (SEM) to test the hypothesized relationships between the latent variables. Model fit was evaluated using the same fit indices as in the CFA. Hypothesized mediation effects were tested using bootstrapping with 5,000 resamples to generate bias-corrected confidence intervals (Preacher and Hayes, 2008). To further explore the potential moderating effect of gender, we conducted a multi-group SEM analysis. This involved a series of hierarchical invariance tests (configural, metric, scalar, and structural) to determine whether the relationships between the variables differed across genders (Byrne, 2016; Cheung and Rensvold, 2002).

## 5 Findings

### 5.1 Data preprocessing

Prior to analysis, data were screened for missing values, outliers, and normality assumptions. Missing data (approximately 3%) were handled using the expectation–maximization (EM) algorithm, which provides unbiased parameter estimates. Little’s MCAR test (χ^2^(32) = 35.21, *p* = 0.29) confirmed that data were missing at random, validating the use of EM for imputation (Little and Rubin, 2019). Outliers were identified through Z-scores (±3.29 standard deviations) for univariate outliers and Mahalanobis distance (*p* < 0.001) for multivariate outliers (Kline, 2016). This process flagged 12 univariate and 8 multivariate outliers. Sensitivity analyses showed minimal impact on results; however, these outliers were excluded to ensure robustness. Normality assumptions were evaluated by examining skewness (−0.25 to 0.35) and kurtosis (−0.45 to 0.82), which indicated univariate normality. Mardia’s coefficient (2.87) confirmed multivariate normality (Byrne, 2016).

To address potential common method bias (CMB) from self-reported measures, several procedural safeguards were implemented, including assurances of anonymity, clear language, and randomized item order to minimize response patterns. Harman’s single-factor test revealed that the first factor accounted for 24% of the variance, below the 50% threshold, indicating minimal CMB (Podsakoff et al., 2003). Additionally, a marker variable unrelated to the main constructs showed no significant correlation (*r* = 0.03), further supporting the absence of meaningful CMB.

### 5.2 Descriptive statistics

Table 2 presents the descriptive statistics for all key study variables, including gratitude, perceived social support, the six dimensions of prosocial tendencies (altruistic, compliant, emotional, public, anonymous, and urgent), and psychological well-being. The prosocial tendencies construct was modeled as a higher-order factor (HOF) in subsequent analyses, reflecting the shared variance among its six dimensions. Descriptive statistics for these dimensions are provided individually in Table 2. A composite score representing the higher-order factor “prosocial tendencies” was calculated by averaging the *z*-scores of the six dimensions.

**Table 2:** Descriptive statistics for study variables.

Variable	*N*	Mean	SD	Min	Max
Gratitude	703	5.30	0.95	2.0	7.0
Perceived social support	703	4.85	0.78	2.5	6.5
Altruistic prosocial tendency	703	3.85	0.90	1.9	5.0
Compliant prosocial tendency	703	3.50	0.85	1.8	5.0
Emotional prosocial tendency	703	3.70	0.88	1.7	5.0
Public prosocial tendency	703	3.25	0.85	1.5	5.0
Anonymous prosocial tendency	703	3.90	0.87	2.0	5.0
Urgent prosocial tendency	703	3.45	0.89	1.6	5.0
Psychological well-being	703	5.15	1.00	2.1	6.9

The mean score for gratitude was 5.30 (SD = 0.95), indicating a relatively high level of gratitude among participants, with scores ranging from 2.0 to 7.0, indicating that most participants reported moderate to high levels of gratitude. Perceived social support had a mean score of 4.85 (SD = 0.78), reflecting a moderate to high level of perceived support. Scores on the perceived social support measure ranged from 2.5 to 6.5. For the dimensions of prosocial tendencies, mean scores varied slightly, with altruistic prosocial tendency showing the highest mean (M = 3.85, SD = 0.90), and public prosocial tendency the lowest (M = 3.25, SD = 0.85). Finally, psychological well-being had a mean score of 5.15 (SD = 1.00), reflecting relatively high levels of well-being. Scores on the psychological well-being measure ranged from 2.1 to 6.9 (see Table 2). The skewness and kurtosis values for all variables, including the dimensions of prosocial tendencies, fell within acceptable ranges (skewness: −0.30 to 0.40; kurtosis: −0.50 to 0.85), indicating approximate normality.

### 5.3 Correlations among study variables

Table 3 presents the correlations among the study variables, including the six dimensions of prosocial tendencies and the higher-order factor (HOF) modeled in the structural equation model. Gratitude was positively correlated with perceived social support (*r* = 0.46, *p* < 0.001) and psychological well-being (*r* = 0.48, *p* < 0.001). Perceived social support showed a strong positive correlation with psychological well-being (*r* = 0.43, *p* < 0.001) and a significant relationship with the prosocial tendencies HOF (*r* = 0.55, *p* < 0.001).

**Table 3:** Correlation matrix for study variables.

Variable	1	2	3	4	5	6	7	8	9	10
1.Gratitude	–									
2.Perceived social support	0.46**	–								
3.Altruistic Prosocial Tendency	0.36**	0.39**	–							
4.Compliant Prosocial Tendency	0.28**	0.34**	0.42**	–						
5.Emotional Prosocial Tendency	0.32**	0.36**	0.45**	0.39**	–					
6.Public Prosocial Tendency	0.24**	0.28**	0.30**	0.35**	0.31**	–				
7.Anonymous Prosocial Tendency	0.31**	0.34**	0.39**	0.30**	0.35**	0.29**	–			
8.Urgent Prosocial Tendency	0.23**	0.27**	0.25**	0.29**	0.23**	0.22**	0.21**	–		
9.Psychological well-being	0.48**	0.43**	0.47**	0.39**	0.44**	0.33**	0.41**	0.29**	–	
10.Prosocial Tendencies (HOF)	0.38	0.55**	0.71	0.65	0.72	0.50	0.68	0.60	0.42**	–

HOF = Higher-Order Factor, *p* < 0.001 for all coefficients.

The six dimensions of prosocial tendencies exhibited varying levels of correlation with psychological well-being. Altruistic (*r* = 0.47, *p* < 0.001) and emotional (*r* = 0.44, *p* < 0.001) tendencies demonstrated the strongest positive associations, while public prosocial tendency showed the weakest positive association (*r* = 0.33, *p* < 0.001). The prosocial tendencies HOF showed moderate to strong correlations with its constituent dimensions, including altruistic (*r* = 0.71, *p* < 0.001), emotional (*r* = 0.72, *p* < 0.001), and anonymous (*r* = 0.68, *p* < 0.001) prosocial tendencies. Overall, the higher-order factor was moderately correlated with psychological well-being (*r* = 0.42, *p* < 0.001).

### 5.4 Confirmatory factor analysis

A confirmatory factor analysis (CFA) was conducted to validate the measurement model, which included the latent variables of gratitude, perceived social support, a higher-order factor for prosocial tendencies, and psychological well-being. The six dimensions of prosocial tendencies (public, anonymous, altruistic, compliant, emotional, and urgent) were specified as indicators of the higher-order factor. This model demonstrated a good fit to the data: χ^2^(180) = 520.35, *p* < 0.001, CFI = 0.93, TLI = 0.92, RMSEA = 0.06 (90% CI [0.05, 0.07]), SRMR = 0.05. The fit indices indicate that the hypothesized model is a good representation of the data. All factor loadings were statistically significant (*p* < 0.001). Specifically, the factor loadings of the prosocial tendencies dimensions on the higher-order factor were as follows: Public (0.71–0.81), Anonymous (0.73–0.85), Altruistic (0.66–0.82), Compliant (0.69–0.79), Emotional (0.65–0.79), and Urgent (0.69–0.84). These CFA loadings confirm that all six dimensions contribute meaningfully to the higher-order construct of prosocial tendencies.

This CFA model with a higher-order factor for prosocial tendencies was chosen over a model with separate latent variables for each dimension due to its greater parsimony and conceptual clarity. While both models demonstrated acceptable fit, the higher-order factor model provides a more concise and interpretable representation of the data by capturing the overall construct of prosocial tendencies while still accounting for the unique contributions of its dimensions.

During the CFA process, several diagnostic checks were performed to assess the potential for model improvement. No modifications were required, as the CFA loadings for all items exceeded the recommended threshold of 0.60 (Kline, 2016), and theoretical considerations supported retaining all items. There was no need to correlate error terms or remove any items, as they aligned well with the constructs measured.

In addition to the prosocial tendencies dimensions, the other latent variables also showed strong relationships with their respective indicators. Specifically, the CFA loadings for gratitude items ranged between 0.70 and 0.82, perceived social support items between 0.68 and 0.83, and psychological well-being items between 0.75 and 0.88. These results confirm the adequacy of the hypothesized factor structure and the strong alignment of items with their respective latent constructs.

To assess the reliability and validity of the measurement model, we examined composite reliability, AVE, and discriminant validity. Composite reliability (CR) estimates exceeded the recommended threshold of 0.70, indicating good internal consistency for all constructs (Fornell and Larcker, 1981). The CR values were 0.83 for gratitude, 0.85 for perceived social support, 0.91 for the higher-order factor prosocial tendencies, and 0.89 for psychological well-being (see Table 4). Additionally, the average variance extracted (AVE) for each construct was greater than 0.50, with AVE values of 0.60 for gratitude, 0.63 for perceived social support, 0.65 for “Prosocial Tendencies,” and 0.64 for psychological well-being, confirming that a substantial amount of variance in the indicators was explained by the latent constructs.

**Table 4:** Confirmatory factor analysis results for measurement model.

Latent variable	Composite reliability	AVE	Factor loadings
Gratitude	0.83	0.60	0.70–0.82
Perceived social support	0.85	0.63	0.68–0.83
Prosocial tendencies	0.91	0.65	Public (0.71–0.81), Anonymous (0.73–0.85), Altruistic (0.66–0.82), Compliant (0.69–0.79), Emotional (0.65–0.79), Urgent (0.69–0.84)
Psychological well-being	0.89	0.64	0.75–0.88

Discriminant validity was assessed by comparing the AVE values to the squared correlations between constructs, following the Fornell-Larcker criterion. In all cases, the AVE for each construct was higher than the squared correlations, supporting discriminant validity (see Table 1). For further confirmation, the heterotrait-monotrait (HTMT) ratios were also examined. All HTMT values were below the conservative threshold of 0.85 (Henseler et al., 2015), providing additional support for discriminant validity.

## 6 Structural relationships

The hypothesized structural equation model (SEM) was tested to assess the relationships between gratitude, perceived social support, a higher-order factor for “Prosocial Tendencies,” and psychological well-being. The six dimensions of prosocial tendencies (public, anonymous, altruistic, compliant, emotional, and urgent) were specified as indicators of the higher-order factor in the model.

To ensure the robustness of our hypothesized model, which includes a higher-order factor for prosocial tendencies, we tested two alternative models for comparison. The first alternative model (Model 1) specified the six dimensions of prosocial tendencies as separate, correlated factors, allowing for greater specificity but also increased complexity. The second alternative model (Model 2) specified a single-factor structure for prosocial tendencies, representing a more parsimonious but potentially less nuanced representation of prosocial behavior.

The hypothesized model, with the higher-order factor, showed a good fit: χ^2^(125) = 378.45, *p* < 0.001, CFI = 0.941, TLI = 0.930, RMSEA = 0.058 (90% CI [0.049, 0.067]), and SRMR = 0.048. The CFI and TLI values exceeded the 0.90 threshold, and both RMSEA and SRMR were within acceptable limits (Hu and Bentler, 1999; Kline, 2016). The fit indices for all three models are presented in Table 5.

**Table 5:** Model fit comparison.

Model	χ^2^	df	CFI	TLI	RMSEA	SRMR
Hypothesized model	378.45	125	0.941	0.930	0.058	0.048
Model 1 (first-order)	450.87	135	0.912	0.898	0.072	0.063
Model 2 (single-factor)	590.25	136	0.863	0.843	0.089	0.071

As shown in Table 5, the hypothesized higher-order factor model demonstrated superior fit compared to both alternative models. Model 1 (first-order factors) showed acceptable but weaker fit (CFI = 0.912, RMSEA = 0.072) and lacked the parsimony of the higher-order model. Model 2 (single-factor structure) exhibited poor fit (CFI = 0.863, RMSEA = 0.089), suggesting that a single factor is insufficient to capture the multidimensional nature of prosocial tendencies.

**Figure 1:**
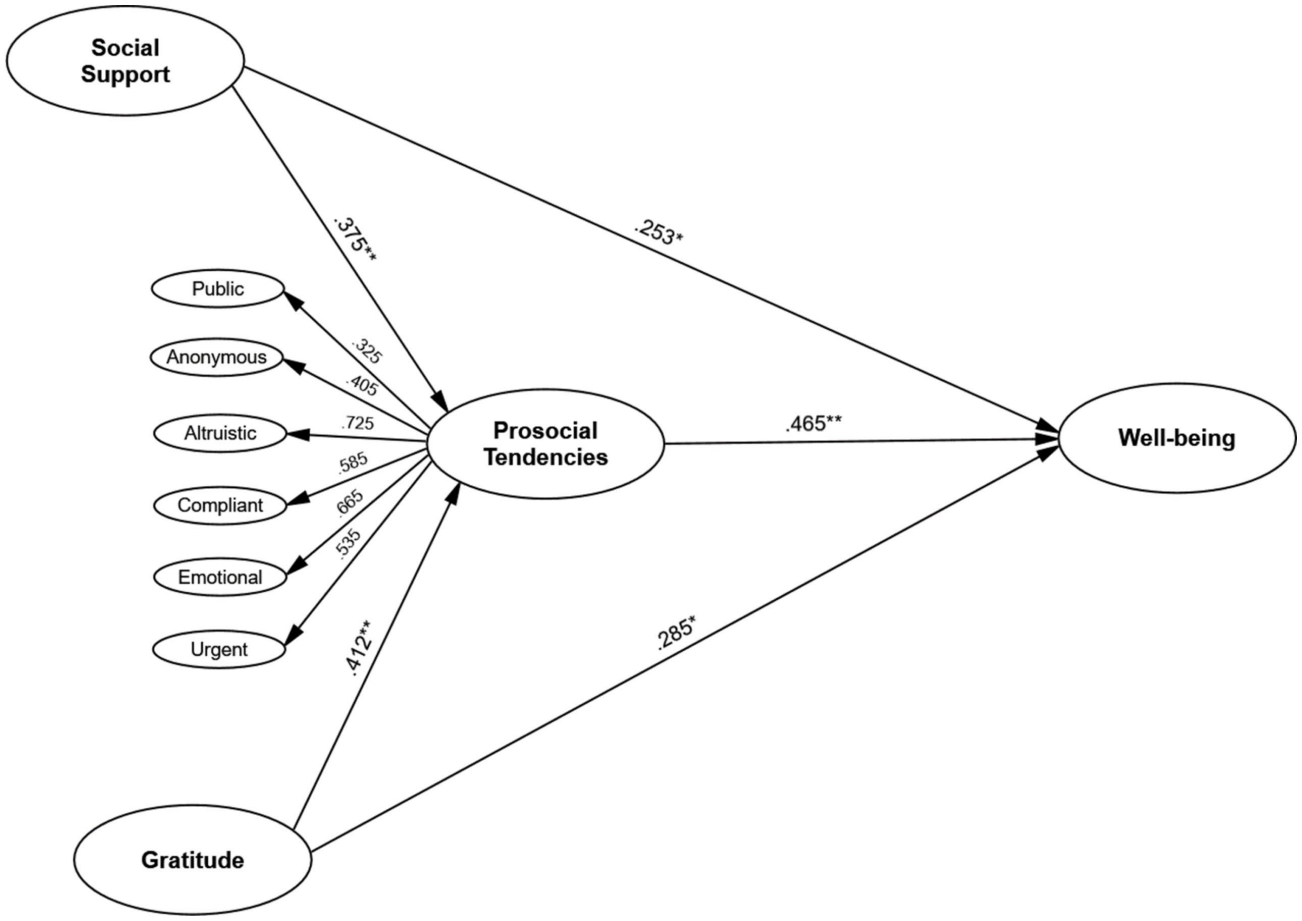
Structural pathways.

A chi-square difference test further supported the hypothesized model as the best-fitting structure (Δχ^2^ = 72.42, *p* < 0.001 for Model 1 vs. the hypothesized model; Δχ^2^ = 211.80, *p* < 0.001 for Model 2 vs. the hypothesized model). Therefore, we retained the hypothesized model with the higher-order factor for prosocial tendencies for all subsequent analyses. This decision is consistent with the principle of parsimony in model selection, which favors simpler models that adequately explain the data without unnecessary complexity (Kline, 2016). Thus, the SEM with the higher-order factor adequately captured the hypothesized relationships among the variables. Figure 1 depicts the significant path coefficients between the constructs, illustrating the strength and direction of the relationships in the model.

All direct effects in the structural model, including the paths from the higher-order factor to its dimensions, were statistically significant (see Table 6), with 95% confidence intervals reported to indicate the precision of these estimates. Gratitude had a positive effect on the higher-order factor prosocial tendencies (*β* = 0.412, SE = 0.058, 95% CI [0.298, 0.526], *p* < 0.001), indicating that individuals with higher levels of gratitude were more likely to engage in prosocial behaviors overall. Similarly, perceived social support also had a positive effect on prosocial tendencies (*β* = 0.375, SE = 0.052, 95% CI [0.273, 0.477], *p* < 0.001), suggesting that individuals who felt more supported by their social networks were more inclined to engage in prosocial behaviors. Gratitude was positively associated with psychological well-being (*β* = 0.285, SE = 0.047, 95% CI [0.193, 0.377], *p* < 0.05), as was perceived social support (*β* = 0.253, SE = 0.051, 95% CI [0.153, 0.353], *p* < 0.05). The higher-order factor prosocial tendencies had a strong positive effect on psychological well-being (*β* = 0.465, SE = 0.053, 95% CI [0.361, 0.569], *p* < 0.001), demonstrating that engaging in prosocial behaviors, as captured by the higher-order factor, contributed significantly to overall psychological health.

**Table 6:** Direct effects in the structural model.

Path	Standardized coefficient (β)	SE	95% CI	*p*-value
Gratitude → Prosocial Tendencies (HOF)	0.412	0.058	[0.298, 0.526]	<0.001
Perceived social support → Prosocial Tendencies (HOF)	0.375	0.052	[0.273, 0.477]	<0.001
Prosocial Tendencies (HOF) → Public	0.325	0.041	[0.405, 0.245]	<0.001
Prosocial Tendencies (HOF) → Anonymous	0.405	0.048	[0.311, 0.499]	<0.001
Prosocial Tendencies (HOF) → Altruistic	0.725	0.062	[0.603, 0.847]	<0.001
Prosocial Tendencies (HOF) → Compliant	0.585	0.051	[0.485, 0.685]	<0.001
Prosocial Tendencies (HOF) → Emotional	0.665	0.059	[0.549, 0.781]	<0.001
Prosocial Tendencies (HOF) → Urgent	0.535	0.049	[0.439, 0.631]	<0.001
Gratitude → Psychological well-being	0.285	0.047	[0.193, 0.377]	<0.05
Perceived social support → Psychological well-being	0.253	0.051	[0.153, 0.353]	<0.05
Prosocial Tendencies (HOF) → Psychological well-being	0.465	0.053	[0.361, 0.569]	<0.001

HOF, higher-order factor.

The mediation analysis, conducted using bias-corrected bootstrap confidence intervals based on 5,000 resamples, examined the indirect effects of gratitude and perceived social support on psychological well-being through the higher-order prosocial tendencies factor (Preacher and Hayes, 2008). As shown in Table 7, the indirect effect of gratitude on psychological well-being via “Prosocial Tendencies” was significant (*β* = 0.195, 95% CI [0.128, 0.262], *p* < 0.001), as was the indirect effect of perceived social support on psychological well-being through prosocial tendencies (*β* = 0.178, 95% CI [0.112, 0.244], *p* < 0.001). This indicates partial mediation, as the direct effects of both gratitude and perceived social support on psychological well-being remained significant even after accounting for the mediator. The higher-order factor prosocial tendencies explained 40% of the variance in psychological well-being, underscoring its critical role as a mediator in this model.

**Table 7:** Indirect effects in the structural model.

Indirect path	Standardized coefficient (β)	95% CI	*p*-value
Gratitude → Prosocial Tendencies (HOF) → psychological well-being	0.195	[0.128, 0.262]	<0.001
Perceived social support → Prosocial Tendencies (HOF) → psychological well-being	0.178	[0.112, 0.244]	<0.001

The total effects, representing both direct and indirect relationships, revealed that gratitude had a substantial overall impact on psychological well-being (*β* = 0.480, SE = 0.071, 95% CI [0.340, 0.620], *p* < 0.001), as did perceived social support (*β* = 0.431, SE = 0.063, 95% CI [0.307, 0.555], *p* < 0.001) (see Table 8). Notably, prosocial tendencies accounted for approximately 41% of the total effect of gratitude on psychological well-being and 41% of the total effect of perceived social support on psychological well-being, highlighting the significant mediating role of prosocial behavior, as captured by the higher-order factor, in these relationships.

**Table 8:** Total effects in the structural model.

Total effect path	Standardized coefficient (β)	Standard error (SE)	*p*-value
Gratitude → psychological well-being	0.480	0.071	<0.001
Perceived social support → psychological well-being	0.431	0.063	<0.001

## 7 Group comparisons

To investigate the potential influence of gender on the relationships between gratitude, perceived social support, the higher-order factor prosocial tendencies, and psychological well-being, a multi-group SEM analysis was conducted (Byrne, 2016; Cheung and Rensvold, 2002). A series of hierarchical invariance tests were performed to assess gender invariance, including configural, metric, scalar, and structural invariance (Cheung and Lau, 2012).

The configural invariance model, which allows for freely estimated parameters across gender groups, was tested first to establish a baseline model. This model demonstrated an acceptable fit (CFI = 0.938, RMSEA = 0.059, SRMR = 0.050), indicating that the factor structure was equivalent across genders. Following this, metric invariance was tested by constraining the factor loadings to be equal for both groups. The fit indices for this model (CFI = 0.937, RMSEA = 0.060, SRMR = 0.051) were similar to those of the configural model, suggesting that the factor loadings were invariant across male and female participants.

Scalar invariance was then tested by further constraining the intercepts to be equal across groups. The scalar model also fit the data well (CFI = 0.936, RMSEA = 0.061, SRMR = 0.052), indicating equivalence in intercepts. Finally, structural invariance was tested by constraining the structural paths between the study variables to be equal across gender. This involved examining the paths from gratitude and perceived social support to the higher-order factor prosocial tendencies, as well as the path from prosocial tendencies to psychological well-being. The structural model demonstrated a good fit (CFI = 0.935, RMSEA = 0.062, SRMR = 0.053), remaining within acceptable limits.

To assess potential differences between the constrained and unconstrained models, changes in CFI (ΔCFI) and RMSEA (ΔRMSEA) were examined. Across all levels of invariance testing, the changes in these indices were minimal (ΔCFI <0.01; ΔRMSEA <0.01), providing strong evidence that the factor structure, factor loadings, intercepts, and structural paths are consistent across gender groups. These results are summarized in Table 9. The invariance findings indicate consistent structural relationships across genders, with no significant differences observed in path coefficients (e.g., gratitude → prosocial tendencies) between male and female participants.

**Table 9:** Model fit indices for gender invariance testing.

Model	χ^2^	df	CFI	RMSEA	SRMR	ΔCFI	ΔRMSEA
Configural	370.25	125	0.938	0.059	0.050	-	-
Metric	374.12	134	0.937	0.060	0.051	0.001	0.001
Scalar	380.85	142	0.936	0.061	0.052	0.001	0.001
Structural	385.98	148	0.935	0.062	0.053	0.001	0.000

The findings from the multi-group SEM analysis indicate no significant gender differences in the structural relationships among the study variables. This suggests that the effects of gratitude, perceived social support, and the higher-order factor prosocial tendencies on psychological well-being are similar for both male and female participants. These results are consistent with prior research, which shows that positive psychological traits and social resources tend to benefit individuals regardless of gender. From a practical perspective, the invariance suggests that interventions aimed at enhancing psychological well-being through gratitude practices, fostering social support, and encouraging prosocial behaviors can be equally effective for both male and female university students. As such, mental health programs on university campuses can implement gender-neutral strategies, as these factors operate similarly across gender groups.

## 8 Discussion

The present study investigated the relationships between gratitude, perceived social support, prosocial tendencies, and psychological well-being among Chinese university students. Drawing from key theories, including Fredrickson’s broaden-and-build theory (2004), Bandura’s social cognitive theory (1986), and Cohen and Wills’ stress-buffering model (1985), the research explored how these psychological factors interact and whether prosocial tendencies mediate the relationships between gratitude, social support, and PWB. The findings provide important insights into how positive emotions, supportive social environments, and prosocial behaviors enhance mental health, particularly in the context of university students facing academic pressures.

The results confirmed that gratitude significantly predicts psychological well-being, consistent with previous research (Watkins, 2013; Wood et al., 2009). According to the broaden-and-build theory, positive emotions like gratitude expand individuals’ cognitive and behavioral options, enabling them to develop lasting personal and social resources (Fredrickson, 2004). In line with this theory, students with higher levels of gratitude reported greater life satisfaction, improved emotional regulation, and better overall mental health. Gratitude fosters personal growth, self-acceptance, and strengthens interpersonal relationships, which are key dimensions of psychological well-being (Ryff and Keyes, 1995; Ryff, 2013). Additionally, gratitude contributes to psychological resilience by helping individuals manage stress more effectively (Portocarrero et al., 2020; Demichelis et al., 2024). This is particularly relevant in educational settings where students face ongoing academic and social pressures. Research shows that gratitude reduces stress and fosters optimism, which enhances emotional health and academic performance (Bono et al., 2022; Deichert et al., 2021). In line with findings from Zhang et al. (2023), our study suggests that gratitude plays a key role in promoting psychological well-being by helping students maintain emotional balance during challenging times.

Perceived social support plays a significant role in promoting psychological well-being, consistent with the stress-buffering model (Cohen and Wills, 1985). The findings of this study align with previous research showing that social support mitigates stress, thereby enhancing resilience and mental health (Wang et al., 2018). University students who perceive strong support from their social networks—whether from family, friends, or significant others—tend to report higher psychological well-being, as social support fosters feelings of belonging, security, and self-worth (Malkoç and Yalçın, 2015). In academic environments, where stress is often driven by exams, coursework, and career uncertainties, social support is essential for maintaining emotional health (Brunsting et al., 2021; Morales-Rodríguez et al., 2020). This buffering effect of social support is particularly important in high-stress situations. Students who perceive greater support are better equipped to regulate their emotions, experience lower levels of anxiety and depression, and maintain overall psychological health (Li and Hasson, 2020; Rehman et al., 2020). These findings reinforce the view that well-being is shaped not only by individual factors but also by the broader social environment and the availability of emotional and practical support.

Our study, which modeled prosocial tendencies as a higher-order factor, revealed that this general prosocial disposition is positively associated with psychological well-being among Chinese university students. This finding is consistent with previous research (e.g., Feng and Zhang, 2021; Haller et al., 2022) and suggests that an overall tendency to engage in prosocial behaviors, regardless of the specific motivation, can contribute to enhanced mental health. Specifically, the higher-order factor of prosocial tendencies was significantly and positively correlated with psychological well-being. These findings align closely with Fredrickson’s (2004) broaden-and-build theory, which suggests that positive emotions arising from prosocial actions broaden an individual’s cognitive and behavioral capacities, enabling them to build personal and social resources over time. This accumulation of resources, in turn, fosters improved psychological well-being by enhancing individuals’ resilience and ability to manage life’s challenges (Fredrickson, 2004; Martela and Ryan, 2016).

Interestingly, our analysis revealed that not all dimensions of prosocial tendencies contribute equally to the higher-order factor, nor do they have the same associations with psychological well-being. While altruistic and emotional prosocial tendencies showed the strongest positive associations with the higher-order factor and with well-being, public prosocial tendencies showed the weakest association with both the higher-order factor and well-being. This finding suggests that the motivation behind prosocial behavior may be an important factor in determining its impact on mental health. It is possible that individuals who engage in prosocial behaviors primarily for public recognition may experience less genuine connection with others or may be more focused on external validation rather than intrinsic satisfaction, which could negatively impact their well-being.

The mediation analysis indicated that the higher-order factor of prosocial tendencies partially mediated the relationships between gratitude and psychological well-being, as well as between perceived social support and psychological well-being. This suggests that an overall prosocial disposition serves as a mechanism through which gratitude and social support enhance psychological well-being. However, the finding that the higher-order factor was only a partial mediator, rather than a full mediator, suggests that other factors beyond prosocial tendencies also contribute to the effects of gratitude and social support on well-being. The fact that public prosocial tendencies showed a weaker association with the higher-order factor and with well-being further highlights the complex interplay between different types of prosocial behaviors and mental health. Future research could explore the specific mechanisms through which different motivations for prosocial behavior may differentially impact well-being, as well as the potential moderating role of individual and cultural factors in these relationships.

An important strength of this study was the examination of gender invariance in the structural relationships among the variables, including the higher-order factor of prosocial tendencies. The multi-group SEM analysis revealed that the structural paths were consistent across genders, indicating that the relationships between gratitude, perceived social support, the higher-order factor of prosocial tendencies, and psychological well-being do not differ significantly between male and female students. This finding suggests that interventions aimed at enhancing well-being through these constructs can be applied universally across genders in the university student population. It aligns with previous research indicating that positive psychological traits and social resources tend to benefit individuals regardless of gender (Yildirim et al., 2023).

One possible explanation for this lack of gender differences relates to cultural norms and evolving social dynamics within the Chinese context. While traditional gender roles have historically influenced behaviors and expectations, there is a growing emphasis on gender equality in education and the workplace (Chen et al., 2020; Ji et al., 2017). This shift may be fostering similar patterns of psychosocial development in both men and women, leading to comparable levels of gratitude, social support, and prosocial behavior. Furthermore, Confucian values, which emphasize harmonious interpersonal relationships and the importance of social connections, are deeply ingrained in Chinese culture (Hwang, 2012; Li and Fischer, 2007). These values may encourage both male and female students to cultivate gratitude, seek social support, and engage in prosocial behaviors as a means of maintaining social harmony and contributing to the collective good. This cultural emphasis on interconnectedness may contribute to the observed gender invariance in the relationships between these constructs and psychological well-being. Moreover, recent research suggests that Chinese young adults, regardless of gender, are increasingly prioritizing personal growth and self-actualization alongside traditional values (Chen et al., 2020). This may further explain the lack of gender differences in the utilization of gratitude, social support, and prosocial behavior to enhance well-being. Both male and female students may be actively seeking these resources to navigate the challenges of university life and achieve their personal goals.

The findings of this study provide clear support for the proposed hypotheses. Hypotheses 1 and 2, which predicted positive associations between gratitude, perceived social support, and psychological well-being, were confirmed. Hypotheses 3 and 4, which proposed that prosocial tendencies would mediate these relationships, were partially supported, with the higher-order factor ‘prosocial tendencies’ serving as a partial mediator in these relationships. This partial mediation suggests that while an overall prosocial disposition is a key pathway through which gratitude and social support enhance well-being, other direct effects also contribute. This finding highlights the complexity of psychological well-being and suggests that interventions aimed at improving mental health should address multiple factors. In line with recent research, our results align with the findings of Wan et al. (2023), who identified a link between prosocial behavior and increased well-being among university students. Similarly, Su et al. (2021) emphasized the role of social support in fostering prosocial behavior, which in turn enhances mental health—a pattern also observed in this study. These parallels with existing research strengthen the validity of our conclusions and underscore the relevance of these constructs in ongoing psychological studies.

In conclusion, this study contributes to a deeper understanding of how gratitude, perceived social support, and a higher-order factor representing prosocial tendencies interact to influence psychological well-being in university students. The results underscore the importance of fostering positive emotions and social connections in promoting mental health, particularly within academic settings. The consistency of these structural relationships across genders suggests that interventions could be applied universally, enhancing both their effectiveness and practicality. These findings carry significant implications for individual-level interventions and educational policies.

## 9 Implications

This study enhances the theoretical understanding of how gratitude, perceived social support, and prosocial tendencies jointly influence psychological well-being. By integrating Fredrickson’s broaden-and-build theory, social cognitive theory (Bandura, 1986), and the stress-buffering model (Cohen and Wills, 1985), it offers a comprehensive framework for interpreting the interactions between positive emotions, social relationships, and prosocial behaviors in relation to mental health.

Fredrickson’s broaden-and-build theory provides a strong foundation for explaining the role of gratitude and prosocial behaviors in fostering well-being. The theory suggests that positive emotions like gratitude broaden individuals’ cognitive and behavioral capacities, encouraging them to engage in prosocial actions that build lasting psychological and social resources (Fredrickson, 2004). The present findings support this theory, as both gratitude and prosocial tendencies were significantly associated with improved psychological well-being. Social cognitive theory (Bandura, 1986) further contributes by explaining the role of perceived social support in shaping behavior and mental health. According to this theory, individuals who feel supported are more likely to engage in prosocial actions because they are confident in their ability to positively affect others. This sense of efficacy enhances feelings of competence and belonging, which are critical components of psychological well-being. The study’s results align with these predictions, showing that perceived social support positively correlates with both prosocial behavior and well-being. The stress-buffering model (Cohen and Wills, 1985) highlights the protective role of social support in mitigating stress’s negative effects on PWB. The finding that perceived social support improves well-being by encouraging prosocial behavior aligns with this model, indicating that supportive relationships provide both emotional resources and motivation for actions that promote psychological resilience.

The practical implications of this study are significant for educational institutions aiming to enhance student well-being by focusing on gratitude, social support, and prosocial behavior. First, the positive link between gratitude and well-being suggests that structured gratitude interventions could be an essential component of mental health initiatives for university students. Universities could implement structured programs such as “Gratitude Week,” where students participate in activities like gratitude journaling, reflective writing, and gratitude-sharing sessions. These activities could be integrated into counseling services, student workshops, or even as part of orientation programs for new students. Research indicates that these exercises help shift focus from stressors to positive experiences, fostering emotional resilience and stability (Zhang et al., 2023; Tolcher et al., 2024). Moreover, universities might consider incorporating gratitude-based training into peer support groups, encouraging students to share strategies for identifying positive aspects of their academic and personal lives. Such peer-led discussions can normalize gratitude practices and help students build lasting habits of appreciative thinking. Classroom activities that incorporate gratitude practices, such as writing thank-you notes or reflecting on positive academic experiences, can help students manage stress, particularly during high-pressure periods like midterms and finals.

Second, the strong association between perceived social support and well-being underscores the importance of developing comprehensive social support systems. Universities should invest in peer mentoring programs that connect students across academic years, allowing senior students to provide guidance and support to their juniors. These programs can be especially beneficial for first-year students, international students, and those experiencing major life transitions, as social integration is critical for emotional well-being (Brunsting et al., 2021). Additionally, mental health services should prioritize community-building initiatives by organizing events that foster connections among students, such as group counseling sessions or social mixers. Faculty members can also play a critical role by creating supportive academic environments, offering mentorship, and maintaining open-door policies to encourage students to seek guidance. Integrating technology-based platforms, such as online support forums or mobile apps, could further expand access to social resources, enabling students to seek help, share experiences, and form supportive networks beyond traditional face-to-face settings.

Third, the findings on prosocial tendencies indicate that promoting prosocial behavior within educational environments can enhance both individual and collective well-being. Universities could establish volunteer programs or service-learning courses that not only provide academic credit but also encourage students to engage in meaningful community service. For instance, organizing monthly community outreach events or partnering with local non-profits can create opportunities for students to contribute positively to society while fostering a sense of purpose. Additionally, peer-led initiatives such as study groups, wellness check-ins, and academic buddy systems can cultivate a culture of compassion and support, leading to improved mental health outcomes (Collie, 2022). Empathy-building workshops, social–emotional learning curricula, and mindfulness training sessions could also help develop prosocial behaviors among students, promoting a more cohesive and engaged academic community. Incorporating regular feedback loops—such as student surveys or focus groups—can help administrators understand which prosocial activities resonate most with students, ensuring that interventions remain relevant, appealing, and impactful.

Finally, specific strategies could be tailored to address challenges faced by different student groups. For instance, first-year students may benefit from structured mentorship and orientation programs, while senior students could engage in leadership roles that encourage prosocial involvement. Faculty training programs aimed at identifying students at risk of isolation or distress can further enhance the institution’s support system. By aligning interventions with cultural values and linguistic preferences—for example, providing workshops or materials in multiple languages—universities can ensure inclusivity and accessibility for diverse student populations. These tailored approaches would ensure that the benefits of gratitude, social support, and prosocial behaviors are effectively translated into practical, impactful interventions across diverse student populations.

## 10 Limitations

This study offers valuable insights into the roles of gratitude, social support, prosocial tendencies, and psychological well-being; however, several limitations should be noted. First, the cross-sectional design prevents causal inferences between variables. Longitudinal studies are needed to examine how changes in gratitude, social support, and prosocial tendencies influence well-being over time, especially during critical academic transitions like the first year of university or final exams. Such designs could also evaluate the long-term effects of interventions targeting gratitude or prosocial behavior.

Second, the use of self-report measures may introduce response biases, such as social desirability bias. Future research should incorporate both self-report and objective behavioral measures to better capture constructs like prosocial tendencies. For instance, experimental designs could assess actual prosocial behaviors in real-world contexts, such as participation in university or community service programs, to validate and extend these findings.

Third, while our study focused on the role of prosocial tendencies in mediating the relationships between gratitude, social support, and psychological well-being, it is important to acknowledge that other potential influencing factors may also play significant roles. Personality traits, such as agreeableness, conscientiousness, and extraversion, have been linked to both prosocial behavior and well-being (Ozer and Benet-Martínez, 2006). Similarly, coping strategies, such as seeking social support, problem-solving, and positive reappraisal, can influence how individuals respond to stress and challenges, which in turn may affect their well-being (Lazarus and Folkman, 1984). Future research could incorporate measures of personality traits and coping strategies to explore their potential roles in the relationships examined in this study.

Fourth, while the study was conducted with a diverse sample of Chinese university students, it is important to consider the cultural context in which the research took place. China is generally considered a collectivist society, where social harmony and group cohesion are highly valued (Triandis, 1995). Therefore, the findings of this study may be most directly applicable to other collectivist cultures. However, the underlying psychological processes of gratitude, social support, and prosocial behavior are likely to be universal, although their specific manifestations and cultural expressions may vary across individualist and collectivist contexts (Emmons and McCullough, 2003). Future research could explore these relationships in diverse cultural settings to examine the generalizability of the findings and identify any culturally specific factors that may influence these processes.

Moreover, although the sample included students from diverse backgrounds, this study did not explicitly explore cultural differences in the relationships between gratitude, social support, prosocial tendencies, and well-being. Future research should investigate how these relationships might vary across different cultural contexts, given that the expression of gratitude and prosocial behaviors can be influenced by cultural norms and values. Cross-cultural comparisons could illuminate how different cultural settings affect the availability and perception of social support and the likelihood of engaging in prosocial behavior. Such insights would allow for more culturally sensitive interventions that are tailored to specific student populations globally.

Fifth, although we conducted a thorough translation and back-translation of our measures, followed by a review by bilingual experts, it is important to acknowledge that a more comprehensive cultural-linguistic validation could further enhance the cross-cultural applicability of our findings. Future studies could employ more rigorous validation methods, such as cognitive interviews and pilot testing with diverse samples, to ensure the cultural equivalence of the instruments.

Finally, future research should also explore the role of other psychological and social variables, such as mindfulness, emotional regulation, and academic self-efficacy, in the proposed model. Integrating these factors could provide a more holistic understanding of the mechanisms driving psychological well-being in university students. Additionally, investigating how academic stress moderates the relationships between gratitude, social support, prosocial tendencies, and well-being could help develop interventions that are responsive to the specific challenges faced by students during high-pressure periods, such as examination times or thesis writing phases.

## Data Availability

The data analyzed in this study is subject to the following licenses/restrictions: the data used and analyzed during the current study are available from the corresponding author upon reasonable request. Requests to access these datasets should be directed to Xu Man, ycxuman@163.com.
